# Modeling and Syndromic Surveillance for Estimating Weather-Induced Heat-Related Illness 

**DOI:** 10.1155/2011/750236

**Published:** 2011-05-04

**Authors:** Alexander G. Perry, Michael J. Korenberg, Geoffrey G. Hall, Kieran M. Moore

**Affiliations:** ^1^Public Health Informatics Group, Kingston, Frontenac, Lennox & Addington Public Health, 221 Portsmouth Avenue, Kingston, ON, Canada K7M 1V5; ^2^Department of Electrical and Computer Engineering, Queen's University, Kingston, ON, Canada K7L 3N6; ^3^Department of Civil Engineering, Queen's University, Kingston, ON, Canada K7L 3N6; ^4^Department of Emergency Medicine, Queen's University, Kingston, ON, Canada K7L 3N6

## Abstract

This paper compares syndromic surveillance and predictive weather-based models for estimating emergency department (ED) visits for Heat-Related Illness (HRI). A retrospective time-series analysis of weather station observations and ICD-coded HRI ED visits to ten hospitals in south eastern Ontario, Canada, was performed from April 2003 to December 2008 using hospital data from the National Ambulatory Care Reporting System (NACRS) database, ED patient chief complaint data collected by a syndromic surveillance system, and weather data from Environment Canada. Poisson regression and Fast Orthogonal Search (FOS), a nonlinear time series modeling technique, were used to construct models for the expected number of HRI ED visits using weather predictor variables (temperature, humidity, and wind speed). Estimates of HRI visits from regression models using both weather variables and visit counts captured by syndromic surveillance as predictors were slightly more highly correlated with NACRS HRI ED visits than either regression models using only weather predictors or syndromic surveillance counts.

## 1. Introduction

The morbidity and mortality associated with extreme heat events such as those in Chicago in 1995 [1], Europe in 2003 [2–4], and California in 2006 [5] underscore the potential impact of heat on population health. Threats of climate change have raised public health concerns over extreme heat events [6]. Preparing for extreme heat events and monitoring their impact is therefore important to public health authorities.

The formulation of public health heat response plans should consider population-level responses to heat in addition to guidelines for safe individual-level heat exposure. Both retrospective analysis of hospital records and real-time surveillance of emergency department visits can provide information on population-level heat response. Each has its benefits and drawbacks.

Abstracted patient charts can provide accurate postdiagnosis identification of heat-related hospital emergency department visits. However, the delays in obtaining data from this source can be over a year due to the time required for abstraction of patient records, reporting, and dissemination. Sometimes only limited historical data are available for analysis. Relying on the results of retrospective analyses to inform heat response plans assumes a population's response to heat does not change over time, neglecting the possibility of long-term acclimation [6]. Responses that occurred historically may not be representative of future responses to heat events if these future events have characteristics that differ from those in the past, such as heat of higher intensities or longer durations [7]. Furthermore, there is evidence that the effects of heat on population health vary geographically [8], raising concerns about the generalizability of study results from other locations. 

To overcome the limitations of an approach using only retrospective analysis to inform public health heat responses, real-time population-level heat-related illness (HRI) surveillance could provide situational awareness, enabling better decisions to be made. However, there is a cost associated with setting up and maintaining such systems, although the economic benefit of heat watch and warning systems has been argued [9]. Heat-related illness surveillance using emergency department (ED) and emergency services dispatch (911) has been suggested [10, 11]. Though not necessarily a comprehensive measure of the complete impact of heat on a population since it fails to capture all care sought for heat illness, heat-related emergency department visits potentially provide a timely indicator of its acute and serious effects. Heat-related mortality has been shown to be correlated with emergency department visits [12]. Since mortality can lag acute heat effects [13] and information on cause of death can take time to be established and disseminated, monitoring emergency department visits would be preferable to monitoring mortality for surveillance purposes. Data from hospitals can often be obtained electronically allowing them to be monitored in real-time using syndromic surveillance [11, 14]. There are unfortunately two potential problems with using emergency department syndromic surveillance. The first is misclassification of visits, a result of nonspecific and nonsensitive syndrome definitions and the use of prediagnosis information. This could lead to errors in visit counts. The second is that such systems do not provide the advance warning required for taking preventative actions. 

Because of the complementary advantages and disadvantages of surveillance and population-level heat-response models constructed from retrospective data on heat-related emergency department visits, public health may consider using both of these approaches. The objective of this study was therefore to investigate and compare syndromic surveillance of heat-related emergency department visits and predictive models for heat-related emergency department visits using weather variables as predictors constructed using retrospective data in south eastern Ontario, Canada, from 2003 to 2008.

A question facing those developing heat response plans is what set of environmental variables or heat measures is best used for issuing warnings and for building models that might predict the expected number of individuals affected by heat-related illness. The direct effects of heat at the level of the individual have been extensively studied, both through physical modeling of heat exchange [15, 16] and physiological studies [17]. These studies indicate that the heat removed through the evaporation of perspiration and the effects of radiant sources of heat (e.g., the sun) play significant roles in the heat stress experienced by an individual. Therefore, in addition to the ambient air temperature, measures of heat exposure should consider the effects of sun, humidity, and wind. A variety of measures of heat exposure that combine these effects have been created and used to develop occupational health and safety exposure guidelines and public health heat warning thresholds including the heat index, humidex (a measure similar to the heat index used in Canada), and wet bulb globe temperature (WBGT) [15, 16, 18]. This study explores the question of which weather variables appear to have population-level significance rather than a priori selecting one of these exposure measures.

Studies of the population-level effects of heat suggest the existence of lagged relationships between heat exposure and illness or mortality, meaning that exposure over and up to several days in advance may impact the development of heat illness or be associated with heat-related mortality [8, 13]. Nonlinear and threshold effects have also been suggested [8, 19]. Population-level acclimation, through mechanisms such as behavioral (e.g., increased use of air conditioning) or physiological adaptation, and survivorship biases may seasonally modify population-level effects of heat [6, 8]. Because of the potential importance of these factors, this study uses nonlinear time series modeling techniques that allow the influence of lagged, nonlinear, and threshold effects of weather variables on heat-related illness to be captured.

To summarize, the objectives of the paper are (1) to construct predictive models for estimating heat-related visits, (2) to develop a simple syndrome definition for syndromic surveillance of heat-related visits, and (3) to compare the predictive models and syndrome definition to a gold standard measure of heat-related visits (NACRS).

## 2. Methods

### 2.1. Study Design

The study used an ecologic retrospective time-series analysis of heat-related hospital emergency department visits to ten hospitals across four health unit jurisdictions in south eastern Ontario, Canada, and weather data from five weather stations in the same area operated by Environment Canada from April 1, 2003 to December 31, 2008. The area covered included Peterborough in the west to Brockville in the east to Bancroft in the north to Lake Ontario in the south. Together these health units had a combined population of approximately 655 000, or roughly 5% of the 2006 Ontario population [20]. Only hospitals in the study area for which emergency department chief complaint data and National Ambulatory Care Reporting System (NACRS) data were available were included.

### 2.2. Outcome Definition and Measurement

Hospital emergency department visits were obtained from the NACRS database. All hospitals in Ontario are required to submit information on emergency department visits to the Canadian Institute for Health Information (CIHI) for inclusion in NACRS. The information in this database includes reasons for the visit, abstracted from patient charts and coded using the International Classification for Disease, 10th revision, Canadian Enhancement (ICD10-CA) coding system. CIHI routinely assesses the quality of data in this database using a number of methods [21]. Because it provides information on the post-diagnosis reason for visit, the NACRS data set was considered to reflect the true reason for visit and therefore served as a gold standard for the actual number of heat-related emergency department visits. Fields used from the NACRS database included ICD-10CA coded reason for visit, institution visited, age, sex, date of visit, partial postal code, and a nonidentifying encounter number that could be used to identify visits for the same individual on the same day, thereby preventing duplicate counting. 

Chief complaint at time of patient registration at the emergency department was obtained for the hospitals included in the study. When an individual presents to the emergency department, the triage nurse enters a short (approximately five to ten word) free-text description of the patient's chief complaint before the patient is diagnosed and treated. This information, visit date and time, age, gender, and partial postal code are sent from hospitals to a central database for use in a real-time syndromic surveillance system. This system has been described in detail elsewhere [22].

Previous epidemiological studies suggest that medical conditions that appear to be associated with hospital visits (admissions as well as to emergency departments) for heat-related illness include not only its direct effects but also malaise [11], some renal conditions [4, 5, 11], electrolyte and fluid imbalance [3, 5, 11], diabetes [5, 11], chronic respiratory conditions, cardiovascular disease, and cerebrovascular disease [4, 5, 8, 23, 24]. Heat has been associated with increased mortality for many of these conditions [4, 13, 23] and physiological evidence substantiates many of these associations [17]. However, because a large proportion of emergency department visits for many of these conditions are likely not heat-related, we defined heat-related illness as only those visits directly attributed to heat: ICD-10CA codes T67.0-T67.9, X30 [10], accepting the limitation that this may underestimate the true number of heat-related emergency department visits.

### 2.3. Exposure Measurement

Many measures of heat exposure have been developed: heat index [15, 25], humidex (Canada) [15, 25], and Wet Bulb Globe Temperature (WBGT) [15]. Because these measures combine different weather variables (some or all of temperature, humidity, wind speed, and solar radiation), and different weightings of each of these variables, this study considered each weather variable separately. This avoided any prior assumptions about the relative importance of individual weather variables in the exposure.

Hourly measurements of weather were obtained from Environment Canada for five weather stations located across Southeastern Ontario near the hospitals included in the study. Measurements included temperature, dew point, pressure, and wind speed. Unfortunately, a direct measure of solar radiation was not available, and therefore this variable was not considered in this study. Maximum, minimum, and average daily values of the temperature, dew point, wind speed, were calculated from the data from each of the five weather stations over the 24-hour period for each day. Missing observations were omitted from these calculations. The average value of the variables across weather stations was used in the analysis. In order to assess the error in doing this, variation in each variable across weather stations was calculated. 

The humidex (HU) and heat index (HI), also referred to as apparent temperature, can be calculated as [15, 25]:

(1) $$\begin{array}{cc} \text{HU} & =T_{a}+0.5555\left(w-10\right),\\ \text{HI} & =-2.719+0.994T_{a}+0.016{\left(D\right)}^{2}, \end{array}$$

where *T*_*a*_ is the air temperature in °C (shielded from ambient radiation), *D* is the dew point in °C, and *w* is the vapour pressure of water in hPa given below. 

Maximum water vapor pressure, *w*, was derived from maximum dew point by

(2) $$w=6.11e^{5417.7530(\left(1/273.16)-(\left(1/273.16+D\right)\right))}.$$

This variable can be considered a transformation of dew point, and may be more directly related to the effects of humidity on an individual's experience of heat [25, 26]. It was considered as a measure of humidity in the analysis.

### 2.4. Analysis

The association between visit counts, maximum temperature, average wind speed, and maximum water vapor pressure were assessed using a Poisson regression model accounting for possible overdispersion using SAS software. Same-day measurements of weather variables were examined in the Poisson regression model; lagged effects were not considered. Interactions were also not considered. Predictor variables in the model included an indicator variable differentiating spring and early summer (April/May/June) from other months of the year to investigate the possibility of acclimation over the period of one year. This choice was informed by preliminary exploratory analyses that had included a separate indicator variable for each month and by the hypothesis that acclimation may affect the response to heat. An indicator variable for weekends was also included in the model to control for the effects of the weekly pattern of emergency department visits [27]. 

Investigating the possibility of lagged and nonlinear relationships, threshold effects, and interactions between weather predictors in modeling their association with heat-related emergency department visit counts requires considering a large number of possible candidate terms in a regression model. To address this challenge, a method developed for nonlinear time-series modeling, Fast Orthogonal Search (FOS), was employed [28] (details of the method are given in this reference and not repeated here). This technique allows the construction of a very flexible nonlinear model describing the relationship between heat-related emergency department visits and weather variables by selecting terms from a potentially very large set of candidate terms. The objective is similar to forward selection in regression, except that the FOS algorithm implicitly expresses the selected candidate terms as a set of orthogonal functions. The reduction in mean square error associated with selecting any given candidate is found without having to actually find the orthogonal functions. FOS also allows the model coefficients for the selected terms to be found without finding these orthogonal functions. This minimizes the required computation and allows a large set of candidate terms (tens to hundreds of thousands) to be rapidly searched, which would otherwise require lengthy computation time. 

The FOS model can be expressed as

(3) $$y\left[n\right]=\overset{M}{\underset{m=0}{\sum }}a_{\text{m}}p_{\text{m}}\left[n\right]+e\left[n\right],$$

where *n* is the time index, *a*_*m*_ is a scalar coefficient, *e*[*n*] represents the error, and *p*_*m*_[*n*] is a selected candidate function. 

The method is potentially useful in examining the relationship between the time series of heat-related emergency department visits and environmental variables because in addition to handling nonlinearity and lagged effects, it allows threshold effects to be included in the candidate set. For example, if we only expect values of temperature over 25°C to be relevant, a function describing a step occurring at that temperature could be included as a candidate. 

In the analysis performed in this study, each candidate was of the general form

(4) $$\begin{array}{cc} p_{m}\left[n\right] & =x_{1}\left[n-q_{11}\right]\ldots x_{1}\left[n-q_{1m_{1}}\right]x_{2}\left[n-q_{21}\right]\\ & \;\ldots x_{2}\left[n-q_{2m_{2}}\right]\ldots x_{p}\left[n-q_{11}\right]...x_{p}\left[n-q_{1p_{m}}\right]\\ & \;\ldots U_{L}\left(x_{1}\left[n-r_{L1}\right]-S_{L1}\right)\ldots U_{R}\left(x_{1}\left[n-r_{R1}\right]-S_{R1}\right)\\ & \;\ldots U_{L}\left(x_{p}\left[n-r_{Lp}\right]-S_{Lp}\right)\ldots U_{R}\left(x_{p}\left[n-r_{Rp}\right]-S_{Rp}\right), \end{array}$$

where *x*_*p*_ is the *p*th predictor. There could be up to *m*_*p*_ factors of a given predictor, *p*, in each candidate function. The lag for each factor of each predictor could be different and is denoted *q*_ij_ where *i* refers to the predictor and *j* refers to each individual factor in that predictor. 

Additionally, step functions, triggered by the value of one of the predictors, were possible factors in a term. The steps allow threshold effects to be modeled. In a right step, denoted *U*_*R*_, when the value of the argument of the step function is *less* than a threshold value, its value is zero; otherwise, it is one. Conversely, for a left step, *U*_*L*_, when the value of the argument of the step function is *greater* than a threshold value, the value is zero; otherwise, it is one. The *S*_*ij*_ parameter in the argument of the step function gives the threshold value of step transition; here the *i* subscript refers to the step type and the *j* subscript refers to the predictor. The value of the predictor used to trigger the step is compared to *S*. The lag of this predictor, *r*_*ij*_, was taken to be the minimum lag allowed in that predictor (i.e., *r*_*ij*_ = min _*k*_ {*q*_*j*1_, *q*_*j*2_,…, *q*_*jm*_*p*__}). Only one step function was allowed per predictor in each candidate term. The FOS algorithm was programmed by the authors using MATLAB, and the implementation was verified by fitting known relationships.

Each candidate term in the model could contain multiple factors. Log-transformed visits were considered as the model output. Predictors included the same weather variables used for the Poisson regression model. Table 1 describes the component factors possible in a given candidate term by giving the limits on the parameter values for (4) for each predictor. Step functions could occur without any factors of the corresponding predictor occurring. In preliminary exploratory analyses, maximum temperature and water vapour pressure lagged at up to seven days were allowed as candidates. The values in Table 1 were selected based on previous literature and exploratory analysis. Visit counts were log-transformed before fitting the model. The set of candidate terms searched included all possible combinations of each of the factors, approximately 140 000 possible candidate terms, from which FOS obtained a concise model within minutes on a standard laptop computer.

### 2.5. Heat-Related Illness Syndrome Definition

To develop a syndrome definition for heat-related illness based on emergency department chief complaint, NACRS visits (the gold standard) were matched to emergency department chief complaint using date and time of visit, hospital visited, partial postal code, age, and gender since a unique identifier was not available for matching records between data sets. By evaluating the positive predictive value (PPV) of the most commonly appearing text strings in emergency department chief complaints relative to the NACRS gold standard, a set of strings identifying likely heat-related visits was created. The resulting time-series of heat-related visits counts was created by counting the daily number of emergency department chief complaints containing at least one of the text strings in the set. This time-series was compared to the NACRS time-series of heat-related visit counts.

### 2.6. Model Evaluation and Comparison

A second model for estimating emergency department visits using FOS which included as predictors both daily counts of various text strings in chief complaints found to be associated with heat-related illness in addition to the weather variables was created. If the surveillance data provides additional benefit beyond the weather variables, then such a model should give better estimates of the actual number of heat-related visits. 

To compare the predictive ability of the three models described above (Poisson regression, FOS model containing weather variable predictors only, and FOS model containing weather variables, and chief complaint text strings as predictors), the data was divided into training and validation data sets. The training data set (April 1, 2003 to December 31, 2006) was used to fit the models, and the validation data was used to assess the model fit (January 1, 2007 to December 31, 2008). Model estimates were compared to the gold standard (NACRS heat-related emergency department visit time-series) using the mean square error (MSE) and Pearson correlation coefficient.

### 2.7. Ethics

Ethics approval for the study was obtained from the Queen's University Health Sciences and Affiliated Teaching Hospitals Research Ethics Board.

## 3. Results

Heat-related emergency department visits ranged from 67 to 161 per year for the complete years included in the study (2004 to 2008). The median age of individuals with heat illness was 29, and half of those presenting to the emergency department were between the ages of 18 and 49. Ten percent were over age 71, and ten percent were under age 11. 

The variation in weather variables across the five weather stations in the study area is given in Table 2. 

Table 3 presents the parameter estimates, associated standard error, and *P* values for the Poisson regression model which included maximum temperature, maximum water vapour pressure, average wind speed, and weekend and spring/early summer indicator variables as predictors of visits. The results show that of the weather variables included in the model, only maximum water vapour pressure and maximum temperature were significant, while average wind speed was not. 

**Figure 1:**
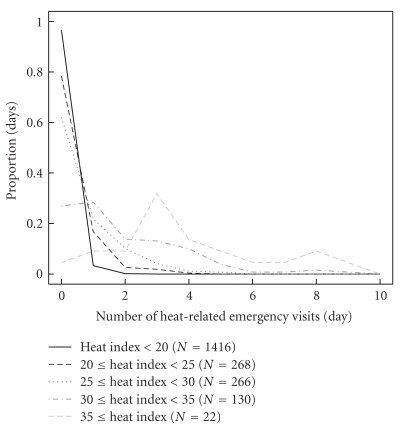
Distribution of the daily number of emergency visits for heat-related illness for five heat index ranges.

Figure 1 shows the proportion of days with a given number of emergency visits for various Heat Index ranges out of the total days over the study period with that Heat Index range. Heat Index was chosen because it is a function of only temperature and humidity, the only weather variables found to be significantly related to heat in the above model. 

Equation (5) gives the model estimating heat-related emergency department visits, *y*(*k*), using weather variables developed using FOS:

(5) $$\begin{array}{cc} & \text{lo}{\text{g}}_{e}\left[y\left(k\right)\right]\\ & \;=1.237\times 10^{-6}U_{R}\left[t_{\max\;}\left(k\right)-15\right]{\left(t_{\max\;}\left(k\right)-15\right)}^{2}\\ & \;\;\times \left(t_{\max\;}\left(k-1\right)-15\right)v\left(k-0\right)v\left(k-1\right)U_{L}\left[s\left(k\right)-20\right]\\ & \;\;+4.356\times 10^{-4}U_{R}\left[t_{\max\;}\left(k\right)-15\right]\left(t_{\max\;}\left(k\right)-15\right)\\ & \;\;\times \left(t_{\max\;}\left(k-1\right)-15\right)U_{L}\left[s\left(k\right)-20\right]g\left(n\right)v\left(k-1\right)\\ & \;\;-4.476\times 10^{-4}U_{R}\left[t_{\max\;}\left(k\right)-30\right]\left(t_{\max\;}\left(k-2\right)-30\right)\\ & \;\;\times \left(t_{\max\;}\left(k\right)-30\right)U_{L}\left[s\left(k\right)-10\right]\left(s\left(k\right)-10\right){\left(v\left(k-1\right)\right)}^{2}, \end{array}$$

where the symbols for the variables in the model above are given in the headings in the first column of Table 1. 

Of the 1 444 519 emergency department surveillance records containing any chief complaint available over the study period, 95 945 (6.6%) visits could be matched to visits in the NACRS database based on the variables available. In this matched dataset, 225 records represented visits for heat-related illness as defined by the ICD10-CA codes given earlier. The most frequently occurring strings were identified from these confirmed heat-related visits. Table 4 presents the number of visits from the matched dataset containing each string, for both visits that were for heat-related illness and not for heat-related illness based on the associated ICD-coded reason for visit. This table is sorted according to the positive predictive value (PPV) of each string. The strings “sun”, “exhaust”, and “heat” had the highest positive predictive value of visits for heat-related illness of the frequently occurring strings and therefore defined the heat-related syndrome. However, using these three strings combined flagged only 81 of the 225 (36%) true visits for heat-related illness, while flagging another 40 visits that were not heat-related. 

When the time-series of counts for each of the 14 most frequently occurring strings were included in a candidate set used by FOS in addition to the weather variables used above, the difference equation found by FOS was:

(6) $$\begin{array}{cc} & \text{lo}{\text{g}}_{e}\left[y\left(k\right)\right]\\ & \;\;=9.3114\times 10^{-7}U_{R}\left[t_{\max\;}\left(k\right)-15\right]{\left(t_{\max\;}\left(k\right)-15\right)}^{2}\\ & \;\;\;\times \left(t_{\max\;}\left(k-1\right)-15\right)v\left(k-0\right)v\left(k-1\right)U_{L}\left[s\left(k\right)-20\right]\\ & \;\;\;+4.2143\times 10^{-4}U_{R}\left[t_{\max\;}\left(k\right)-15\right]\left(t_{\max\;}\left(k\right)-15\right)\\ & \;\;\;\times \left(t_{\max\;}\left(k-1\right)-15\right)U_{L}\left[s\left(k\right)-20\right]g\left(n\right)v\left(k-1\right)\\ & \;\;\;+1.359\times 10^{-3}U_{R}\left[t_{\max\;}\left(k\right)-15\right]\\ & \;\;\;\times U_{L}\left[s\left(k\right)-20\right]{\left(v\left(k\right)\right)}^{2}\left(w_{\text{heat}}\left(k\right)\right), \end{array}$$

where *w*_heat_(*k*) is the time-series of counts of emergency department chief complaints containing the string “heat” and the other symbols are again defined in the headings of Table 1. Note that this was the only string of the 14 included in the candidate set found by FOS to reduce the model MSE when weather variables were also included in the model.

Table 5 compares the various methods for estimating heat-related visits counts presented above over the validation time period. The FOS-derived models produced slightly better estimates than the regression model over both training and validation data sets, with the best model for estimating the true visits being the model which included both emergency department chief complaint string count time-series and weather variables as predictors.

**Figure 2:**
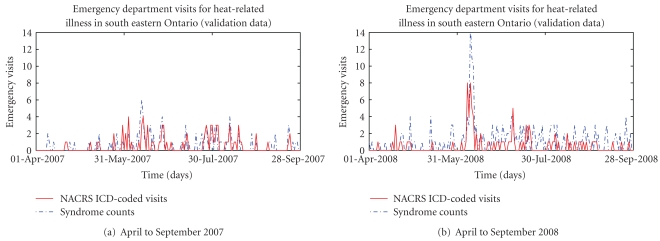
Comparison of syndrome counts for heat-related emergency department visits and NACRS ICD-coded heat-related emergency department visits.

**Figure 3:**
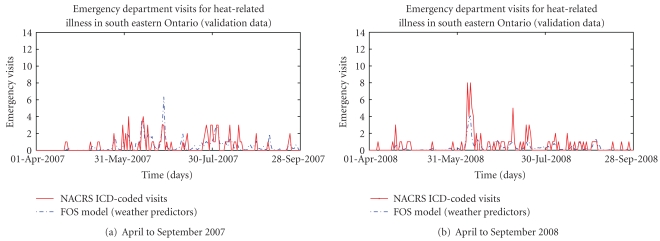
Comparison of estimated visits for heat-related illness from FOS-generated model using weather variables as predictors and NACRS ICD-coded heat-related emergency department visits over validation data.

**Figure 4:**
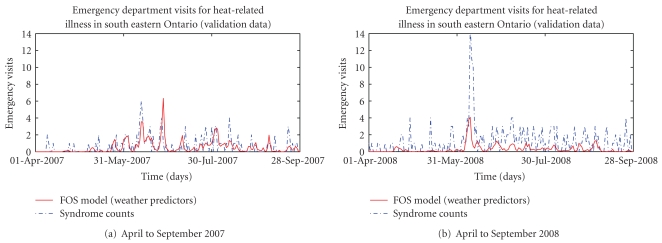
Comparison of syndrome counts for heat-related emergency department visits and estimated heat-related emergency department visits using FOS-generated model with weather-variable predictors over validation data.

**Figure 5:**
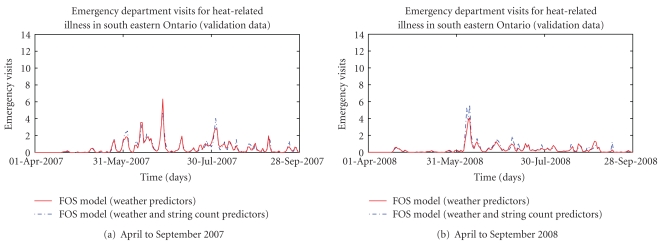
Comparison of FOS generated models using weather predictors only and weather predictors and key string counts for estimating heat-related emergency department visits.

Figures 2, 3, 4, and 5 present provide additional visual comparisons of the time series of the syndrome counts and FOS models to each other and the gold standard NACRS ICD-coded heat-related emergency department visits over the time period used to validate the models.

## 4. Discussion

The results of the regression model indicate that temperature and humidity are most useful in estimating emergency department heat-related visits, although wind speed also appears predictive in the better-performing FOS model. These findings make sense when considering the fact that individuals are more likely to be exposed to humidity and temperature which can be present even indoors compared to wind which can easily be shielded by physical barriers and are more likely to be highly variable across a given geographic region. Inaccurate measurement of wind by insufficiently capturing its variation across the study region may have contributed to this result. Analogously, it may be that heat exposure measures that include variables that have high variability across individuals and or geography, and therefore cannot be adequately measured, may not provide any better measure of population risk of heat-related illness than simpler measures that omit these variables. Unfortunately, this study did not have precise enough measures of solar radiation to allow this variable to be assessed. 

The results of this study suggest the possibility of an “early heat effect”, whereby heat experienced in the spring or early summer (defined as April, May, June) results in more visits. This is consistent with acclimation and/or adaptation effects suggested by other studies [8, 13, 17]. Public health authorities might take this into account in planning and when issuing warnings.

The FOS-derived difference equation models ((5) and (6)) provide better fit than the Poisson regression model over *both* the training and validation data. This suggests that the FOS models are not simply over-fitting the data. However, it is much more difficult to interpret these models as they have terms that consist of interactions of many predictor variables. The model terms in (5) and (6) suggest that the lagged effects of heat on heat-related emergency department visits are relatively short (one day), consistent with findings for hospital admissions [4] but in contrast to the lag suggested for mortality [8, 13]. Note that the first two terms in both models are the same (with the exception of their coefficients which are similar in magnitude). The presence of right step functions in temperature and squares in temperature and humidity are consistent with the threshold and nonlinear effects suggested by other studies [8, 13]. The left step function in wind that appears in the first two terms in both equations, which has positive coefficients in both models, makes sense in that we might expect a higher number of visits when wind speeds are low if wind has a cooling effect. 

The desire to uncover complex relationships between the weather variables and heat related emergency department visits, including threshold, nonlinear, and weather variable interactions, motivated the use of FOS. Testing all of the lagged and interaction terms using standard regression models and techniques would have been extremely tedious at best, and the use of FOS provided a method to check that important relationships were not being missed. However, the difference in performance of this type of model compared to standard regression techniques likely does not justify its use. In practice, simpler regression models may provide similar performance with judicious selection of the proper form of the predictor variables. 

The results of this study suggest that both predictive models using weather variable predictors and syndromic surveillance can provide estimates of heat-related emergency department visits. The small improvement in predictive model performance ((6) versus (5)) after including syndromic surveillance information suggests that measuring the latter may provide additional information not provided by the weather predictors. Since emergency department chief complaint is entered before patients have been examined by a physician and is a brief unstructured description, the syndromic chief compliant data represents a potentially nonsensitive and nonspecific source of information for heat-related emergency department visits. The results of attempting to create a syndrome definition for heat show that it is difficult to derive strings that accurately identify heat-related emergency department visits. Many of the strings appearing in chief complaint for heat-related emergency department visits have low positive predictive value, and those terms that have high enough values to make them useful in a syndrome definition only appear in a small fraction of the visits. It is therefore not surprising that the time series of heat-related visits generated by these terms is noisy, as seen when comparing the heat-related illness syndrome time-series to the NACRS heat-related visits time series. Increased performance might be achieved by using a more sophisticated classifier [29] to flag probable heat-related visits rather than using a simple set of strings as was done here. Failure to use such algorithms in this study is a limitation that makes it difficult to compare the accuracy of model-estimated heat-related emergency department visits with visit counts captured by syndromic surveillance.

Another major limitation of this study was potential exposure misclassification. Weather variables were averaged across weather stations. Ideally, the relationship between weather variables would have been performed for each region separately for maximum exposure accuracy. However, this would have resulted in very few counts in each stratum of the analysis. Table 2 suggests misclassification of wind may have been most significant.

Another weakness of the assessment of the relationship between heat and emergency visits performed in this study was failure to include covariates known to modify the effects of heat (such as age) or underlying medical conditions (such as diabetes), which may vary spatially. This may also bias the observed effect of heat. While these limitations impact the generalizability of the results, they do not impact the study objectives which rely on comparisons within the same population. 

A perfect measure for the true number of heat-related emergency department visits was not available and the amount of error present in the NACRS data is not measurable. It has been suggested that some administrative data sources may underestimate heat-related cases: cases are underreported either because of failure of the clinicians assessing patients to recognize heat as a cause, failure to record this on patient records, or a failure to code heat as an underlying cause when medical records are being abstracted for inclusion in health databases such as NACRS [25, 30]. Since we defined heat-related emergency department visits as only those heat-specific ICD-10CA codes, it is likely that we underestimated the true number of visits. This may explain the low number of heat-related emergency department visits seen in the results of this study. To overcome these problems, other researchers have examined the excess numbers of deaths with heat [25] calculated by removing visits for known sources variability from visit totals; however, we chose to be conservative rather than run the risk of over-estimating numbers. In addition to missing visits, it was also possible that we included some visits not related to environmental heat exposure: the T67 group of ICD codes may include exposure to nonnaturally related heat sources (e.g., occupational exposure), providing a possible explanation for visits seen during winter months. Because of the restricted outcome definition of heat-related illness used in our study, the results may represent only the “tip of the iceberg” of heat effects on population. 

Only a small number of chief complaints could be matched with the NACRS data (less than 7%), and some of these visits may have been mismatches since unique individual identifiers were not available. Difficulty matching and the fact that heat-related visits were relatively rare, reduced the amount of data available for creating a syndrome definition. Because of the lack of data, the syndrome definition was not validated on a set of data different from the one used to create it. This impacts the internal validity of the study: specifically the performance of the heat-related illness syndrome definition may be even less accurate than reported in Table 5. However, there would be no impact on validity of the model given by (6) since all strings in Table 4 were considered equally when constructing the model using FOS which was subsequently validated over a separate data set.

## 5. Conclusion

Temperature and humidity and were significantly associated with increased heat-related emergency department visits while there was less evidence supporting an association with wind speed. Heat in the spring and early summer appear to be associated with more visits, possibly due to subsequent acclimation. Very short lags (0 and 1 day) appeared to be important in explaining visits. Even with potential measurement errors in weather variables, time series models using weather variable predictors, fit using regional historic data, can be constructed that are highly correlated with future emergency department visits not used to fit the models. These models could be used with regional weather forecasts to predict visits and therefore could serve as an evidence-based population risk indicator for issuing heat warnings. Syndromic surveillance of heat-related illness might complement predictive models for estimating and monitoring population-level heat-related illness.

## Figures and Tables

**Table 1:** Parameter ranges and values for candidate terms in FOS model.

Predictor, *p* (symbol)[units]	Maximum factor multiplicity, *m*_*p*_	Maximum lag, max _*k*_ {*q*_*pk*_}	Minimum lag min _*k*_ {*q*_*pk*_}	Step functions: right (*U*_R_), and left (*U*_L_)	Value of predictor at left step function transition, *S*_*Lp*_	Value of predictor at right step function transition, *S*_*Rp*_
maximum temperature (*t*_max_)[°C]	3	2	0	*U*_R_	—	10, 15, 20, 25, 30
Average wind speed(s)[km/h]	1	0	0	*U*_R_, *U*_L_	0, 5, 10, 15, 20	0, 5, 10, 15, 20
spring/early summer indicator (g)	1	0	0	None	—	—
Water vapour (v)[hPa]	2	2	0	None	—	—
Weekend indicator (w)	1	0	0	None	—	—

**Table 2:** Difference in daily weather station measurements across study area.

	Difference in weather station measurements	
	Median	Interquartile range
Maximum temperature (°C)	3.3	2.1
Minimum temperature (°C)	5.9	3.5
Average wind speed (km/h)	8.2	5.4
Maximum dew point (°C)	3.2	2.1

**Table 3:** Poisson regression model parameter estimates.

Poisson regression model			
Parameter	Estimate	Standard error	Parameter significance
Intercept	−7.1591	0.2065	(*P* < .0001)
Water vapour pressure	0.0740	0.0098	(*P* < .0001)
Weekend	0.1378	0.0642	*P* = 0.0319
Maximum temperature	0.2002	0.0115	(*P* < .0001)
April/May/June	0.8196	0.0595	(*P* < .0001)
Average wind speed	−0.0081	0.0092	*P* = .3785

**Table 4:** Positive predictive value of chief complaint text strings associated with heat.

String	Counts in heat-related visits	Counts in nonheatrelated visits	PPV(%)
Sun	39	15	72.2
Exhaust	32	15	68.1
Heat	10	10	50.0
Burn	17	196	8.0
Headache	11	850	1.3
Nausea	9	1127	0.8
Faint	4	488	0.8
Vomit	17	2334	0.7
Syncope	19	4194	0.5
Fever	7	1553	0.4
Fatigue	4	981	0.4
Weak	13	5483	0.2
Dizz	13	5525	0.2
Lighthead	1	424	0.2

**Table 5:** Comparison of syndrome counts and model-based estimates of emergency department visits over the validation time period (January 1, 2007 to December 31, 2008) (error and correlation compared to NACRS ICD-coded heat-related emergency department visits).

	Training data		Validation data	
	Correlation	MSE	Correlation	MSE
Syndrome counts time series	0.57	0.66	0.59	1.06
Poisson regression model	0.76	0.38	0.63	0.37
FOS model (weather predictors)	0.79	0.32	0.66	0.34
FOS model (weather and string count predictors)	0.82	0.30	0.73	0.29
